# Cemento-Ossifying Fibroma of Upper Lip in a Female Child

**DOI:** 10.7759/cureus.14692

**Published:** 2021-04-26

**Authors:** Satish Kumar, Pavithra Dorairaju, V Abirami, Nadeem Jeddy

**Affiliations:** 1 Oral and Maxillofacial Surgery, Thai Moogambigai Dental College and Hospital, Chennai, IND; 2 Oral and Maxillofacial Pathology, Dr. M.G.R. Educational and Research Institute, Chennai, IND; 3 Oral and Maxillofacial Pathology, Thai Moogambigai Dental College and Hospital, Chennai, IND

**Keywords:** cemento-ossifying fibroma, fibro-osseous lesion, neoplastic lesion, benign lesion, odontogenic tumor

## Abstract

Cemento-ossifying fibroma (COF) is a benign tumor classified under fibro-osseous lesions characterized by the proliferation of fibrous tissue associated with the presence of osteoid or cementicle-like masses. COF of bony origin is highly neoplastic in nature compared to their soft tissue counterparts which are relatively rare. The authors here present a case report of COF arising from the left upper lip in a 10-year-old female patient. The lesion was initially asymptomatic, slow-growing in nature, and later turned painful over a period of eight months. A medical CT was taken to elicit a calcified mass seen at the left subcutaneous plane of the upper lip. The lesion was surgically treated by complete excision under local anaesthesia and sutured. Follow-up was done for a period of one year to assess for recurrence which was not evident in this case. This case report, being a peculiar case of COF arising from the soft tissue of the upper lip, describes the clinical presentation, diagnostic imaging, histopathological evidence, and brief surgical management of the lesion.

## Introduction

Cemento-ossifying fibromas (COF) are well-circumscribed, generally slow-growing, benign lesions of the jaws [1]. The tumor consists of proliferative fibrous tissue that is often well vascularized and is characterized by the presence of rounded cementum-like or osteoid bodies, either solitary or together with trabeculae. The exact pathogenesis of COF remains unknown and is often attributed to congenital disturbance during odontogenesis. Radiologically, COF appears as a radiolucent lesion initially and later turns into a radiopaque lesion on maturation [2]. But the localization of COF apart from the jawbones, at the extracranial sites and cranial bones, has given rise to discussions regarding the real nature of cementum-like deposits that are evident in the calciﬁed sections. These pathologic conditions can be categorized as developmental lesions, reactive or dysplastic diseases, and neoplasms [3].

## Case presentation

A ten-year-old female patient reported to the author’s clinic with a complaint of painful swelling on the left upper lip region. The patient had first noticed the swelling eight months back, which was initially asymptomatic and gradually increasing in size. The patient later developed pain in the region of swelling over the last two months. The pain was dull and continuous in nature. The patient had no known underlying medical conditions. Family history revealed no significant finding.

Extra-oral examination revealed a marked asymmetry, with a swelling arising from the left side of the upper lip at the region of canine fossa. The overlying skin appeared normal. The swelling was hard in consistency, had a normal temperature, was tender on palpation and the overlying skin was pinchable. There was no paraesthesia or lymphadenopathy.

**Figure 1:**
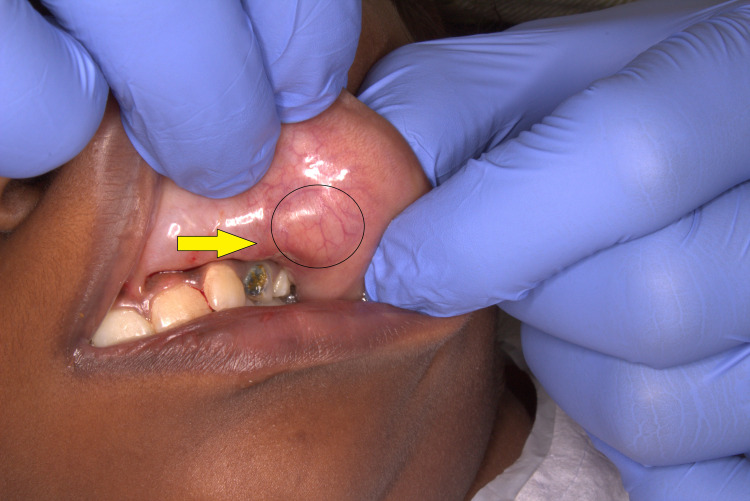
Intra-oral view showing the swelling on the mucosa of the left upper lip (lesion encircled in black and denoted by the yellow arrow).

On intra-oral examination, the swelling appeared ovoid in shape, measuring approximately 1.5 cm x 1 cm. The overlying mucosa appeared normal with no secondary changes (Figure 1). On palpation, the mass was hard in consistency and was not adherent to the underlying tissues. The patient elicited tenderness on palpation. 

**Figure 2:**
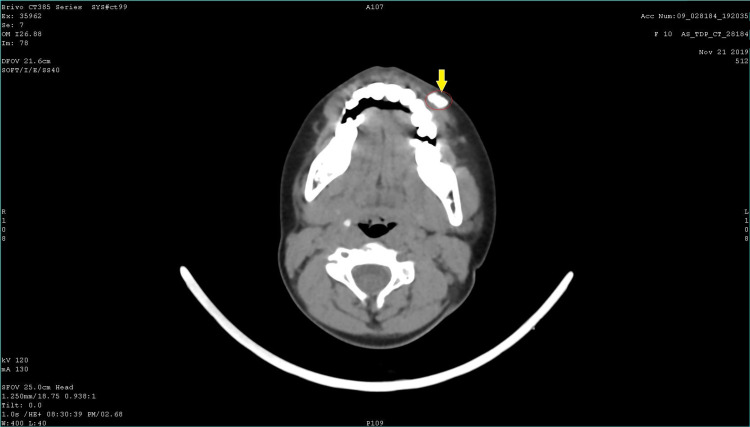
CT axial section showing a calcified mass at the region of the left upper lip (lesion encircled in red and denoted by the yellow arrow).

CT images revealed a well-defined calcified mass of size 11 mm x 6.2 mm x 10 mm seen on the left subcutaneous plane of the upper lip and extending into the adjacent muscular plane. There was no evidence of communication with the maxilla. No adjacent inflammatory changes were seen (Figure 2).

**Figure 3:**
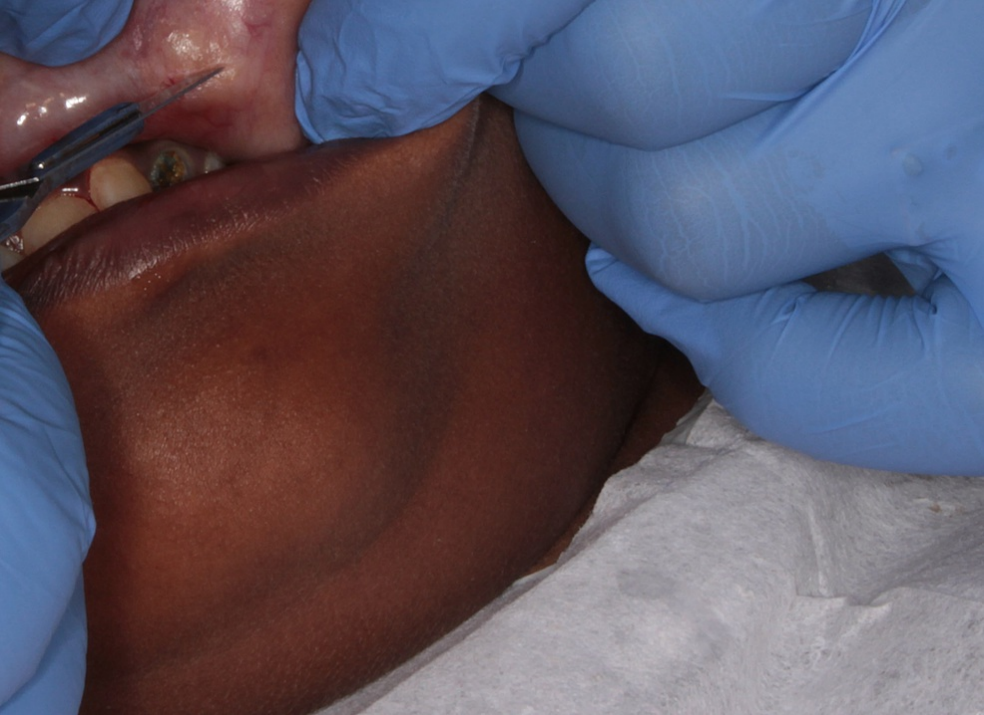
Incision placed on the mucosa overlying the lesion.

**Figure 4:**
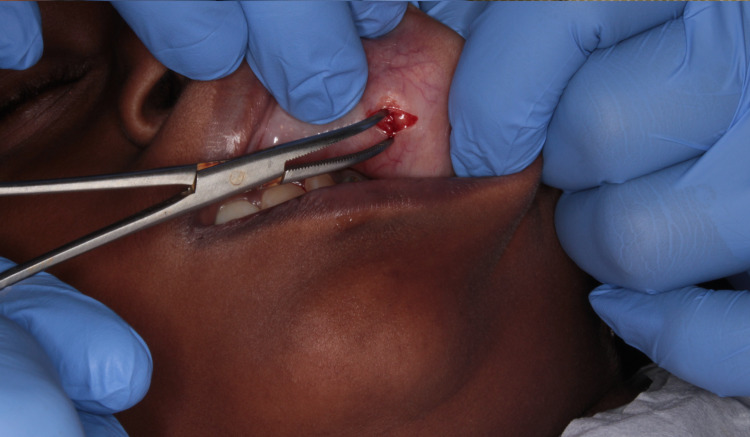
Blunt dissection performed to relieve the lesion.

**Figure 5:**
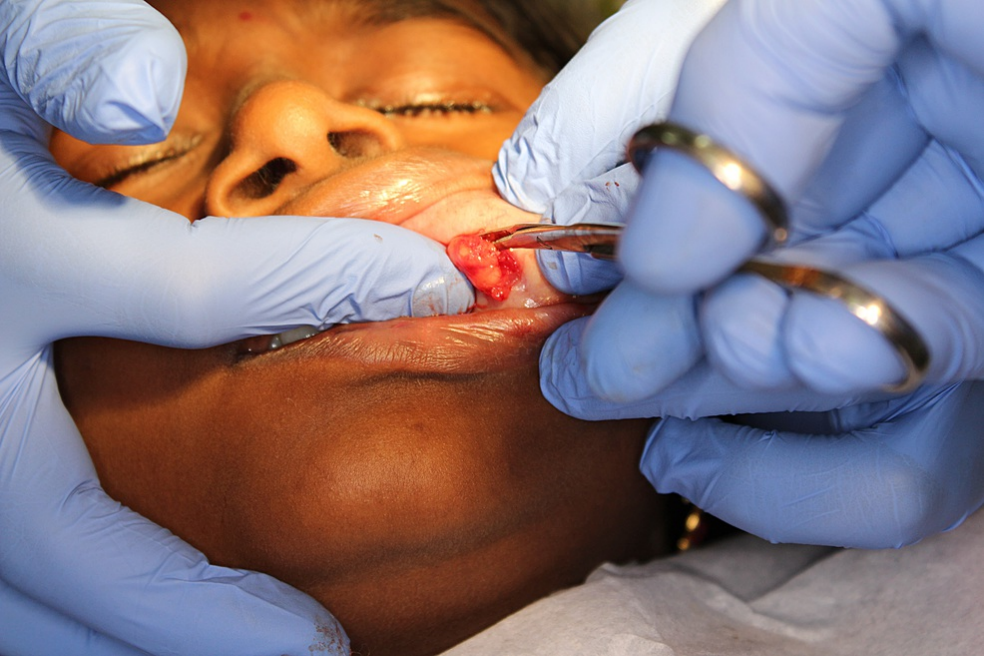
Complete excision of the lesion.

**Figure 6:**
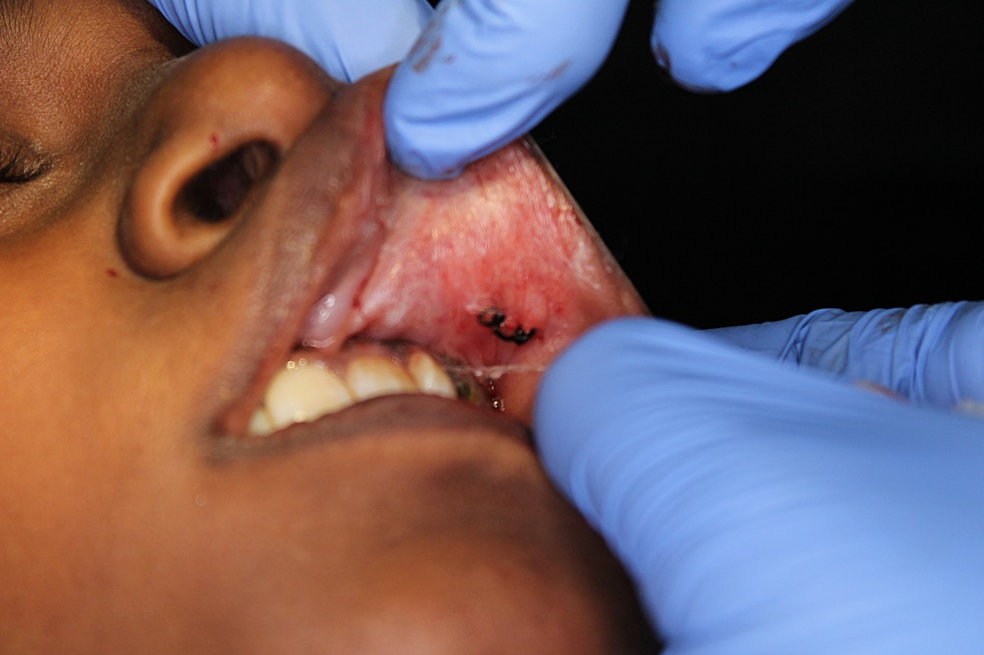
Wound closure is done by suturing using 3-0 braided silk thread.

The management involved the surgical excision of the lesion in toto under local anesthetic solution containing 2% lidocaine HCl with vasoconstrictor (1:100,000 adrenaline). The lip was retracted and the lesion was fixed by pressing a finger against the base of the tumor. An incision was placed over the labial mucosa covering the lesion (Figure 3). Once the muscle layer was reached, blunt dissection was performed to relieve the lesion from the muscle (Figure 4). The lesion was completely excised (Figure 5) and placed in 10% formalin solution and sent for histopathological examination. The wound was sutured using 3-0 black braided silk suture material (Figure 6).

The excised lesion was elliptical in shape and was 1 cm x 0.6 cm x 1 cm in size. It was greyish white in color, hard in consistency, and gritty in texture. The tissue was covered entirely by a capsule. 

**Figure 7:**
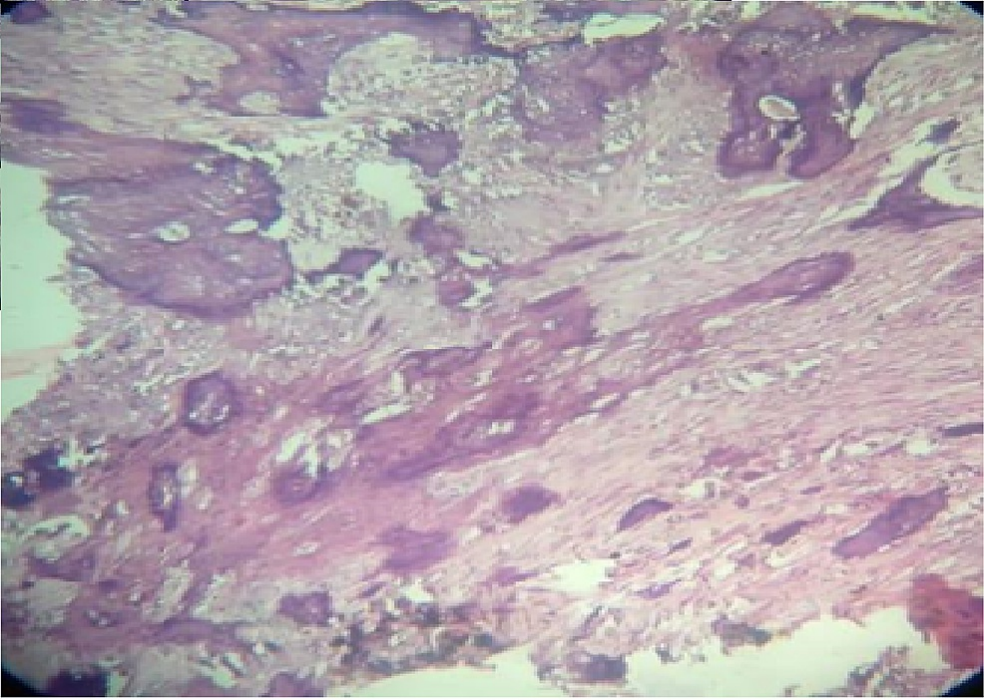
Photomicrograph showing cellular fibroblastic stroma containing spherical ossicles (H and E stained section, ×10 original magnification). H and E: Hematoxylin and Eosin.

Microscopic examination of the Hematoxylin and Eosin stained section revealed a well-circumscribed and fully encapsulated ﬁbro-osseous lesion characterized by abundant cellular ﬁbrous tissue with scattered trabeculae of lamellar bone and spherical ossicles. These ossicles exhibited varying degrees of calcification. These psammoma bodies like ossicles were acellular with a concentric lamination. The lesion is suggestive of a psammomatoid variant of juvenile ossifying fibroma. No cellular atypia or dental tissues were present (Figure 7).

**Figure 8:**
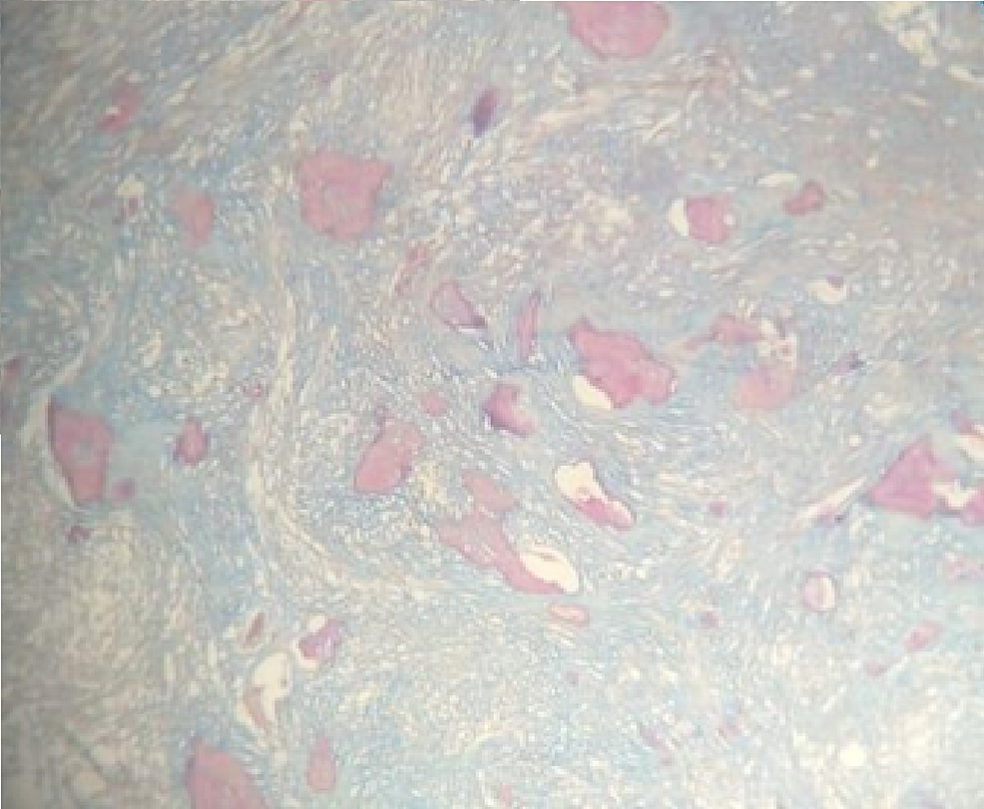
Photomicrograph of a cemento-ossifying fibroma stained with modified Gallego’s stain demonstrating cementum-like deposits in red color (x10).

Modified Gallego's staining of the decalcified section revealed cementum-like deposits in red color (Figure 8).

The patient was recalled after seven days postoperatively. The suture was removed and the surgical site was well irrigated with normal saline. The site appeared to be healing well and the patient did not report any sign of discomfort. The patient was recalled after one month and the surgical site was completely healed. Follow-up was done at periods of three months, six months, and one year. There was no evidence of recurrence of the lesion after one year.

## Discussion

Peripheral COF is a focal, reactive, non-neoplastic tumor-like growth of soft tissue. Cementifying fibromas may be clinically and radiographically impossible to separate from ossifying fibromas [4]. It is widely believed that the cementicles arise from the periodontal ligament [5], while its presence in ectopic regions has often led to debates regarding the pathogenesis of COF. All the more, COF in an atypical site arising from soft tissue is extremely rare. WHO (2005) deﬁned cementum as a mineralized material covering the root surface of the teeth and outside this location, its distinction from bone is equivocal and out of clinical relevance. In 1999, Kaufmann et al. reported a case of COF of the auricle in a 22-year-old man at the region of the right cavum concha, growing in size over a period of three months, the specimen was non-capsulated though well-demarcated [6] unlike this case. In the same year, Jung et al. reported a case of COF in the masticatory and parapharyngeal space with CT and MRI showing extraosseous mass with central conglomerated, well-matured ossified nodules and fatty marrow [7]. COF is usually seen in the second decade of life [8] as a slow, expansive, well-defined mass, unlike other fibro-osseous lesions that are aggressive in nature. The other sites of origin of atypical COF were the petromastoid region, ethmoidal air sinus, and skull base [5,9,10]. Subsequently, in 2009, Longobardi et al. reported a case of COF in the bulk of the cheek [11]. 

The possible differential diagnosis to this case report includes myositis ossificans, traumatic fibroma, and localized osteosarcoma. Myositis ossificans have a histological feature of central fibroblastic tissue, followed by a zone of trabeculae and a peripheral zone of calcification with lamellar bone which is absent in this case. The possibility of traumatic fibroma was excluded due to the absence of any clinically evident stimulus or injurious habit to confirm the same apart from the histopathological differences. Osteosarcoma is undoubtedly ruled out due to the benign nature of the lesion that was histologically confirmed. COF is a peculiar benign lesion that presents with calcified masses of apparently odontogenic origin nevertheless, it can also arise from ectopic regions.

Peripheral COF, per se, is a slow progressing lesion, with limited growth. It has a recurrence rate of 8% to 20%, thereby necessitating the removal of the lesion in toto [12]. A follow-up was done up to one year after the surgery and the patient was clinically assessed for any evidence of recurrence which was negative.

## Conclusions

The reported lesion has a definitive diagnosis of peripheral COF with the histopathological examination under H/E staining revealing fibrous stroma with ossicles. Furthermore, Gallego's staining confirmed the presence of cementicles ruling out the possibility of traumatic fibroma or myositis ossificans. Histopathology showed no presence of giant cells in connective tissue stroma, thus ruling out the possibility of peripheral giant cell granuloma.

This case report demonstrates the rare occurrence of COF in the upper lip and sequential treatment plan elaborating on the investigations, histopathology, and the management of the lesion with a follow-up for one year revealing no evidence of recurrence.
